# Supplementary material for: Modelling Skylarks (Alauda arvensis) to Predict Impacts of Changes in Land Management and Policy: Development and Testing of an Agent-Based Model
Source: PLoS One. 2013 Jun 6;8(6):e65803. doi: 10.1371/journal.pone.0065803 (PMC3675089; doi:10.1371/journal.pone.0065803)
Supplement: Supporting Information S4 — The skylark ODdox as a zipped archive. (ZIP) [file pone.0065803.s004.zip › Skylark_ODdox/class_skylark___nestling.html]

ALMaSS Skylark ODdox: Skylark\_Nestling Class Reference


ALMaSS Skylark ODdox
 2.0


- Main Page
- Related Pages
- Classes
- Files

- Class List
- Class Index
- Class Hierarchy
- Class Members

Public Member Functions |
Protected Member Functions |
Protected Attributes

Skylark\_Nestling Class Reference

`#include <skylarks_all.h>`

List of all members.

|  |  |
| --- | --- |
| Public Member Functions | |
| virtual void | BeginStep (void) |
| virtual void | EndStep (void) |
| virtual double | On\_FoodSupply (double food) |
| void | OnDadDead () |
| void | OnDeserted () |
| void | OnYouHaveBeenEaten () |
|  | Skylark\_Nestling (int x, int y, Skylark\_Male \*Daddy, Landscape \*L, SkTerritories \*Terrs, Skylark\_Population\_Manager \*SPM, int bx, int by, int mh) |
| virtual void | Step (void) |
| Public Member Functions inherited from Skylark\_Base | |
| void | AddStriglingMort (int lifestage) |
| bool | InSquare (rectangle R) |
|  | Skylark\_Base (int x, int y, SkTerritories \*Terrs, Landscape \*L, Skylark\_Population\_Manager \*SPM, int bx, int by, int mh) |
| virtual int | WhatState () |
| Public Member Functions inherited from TAnimal | |
| void | CheckManagement (void) |
| void | CheckManagementXY (int x, int y) |
| virtual void | CopyMyself () |
| virtual void | Dying () |
| virtual void | KillThis () |
| void | SetX (int a\_x) |
| void | SetY (int a\_y) |
| int | Supply\_m\_Location\_x () |
| int | Supply\_m\_Location\_y () |
| unsigned | SupplyFarmOwnerRef () |
| int | SupplyPolygonRef () |
| AnimalPosition | SupplyPosition () |
|  | TAnimal (int x, int y, Landscape \*L) |
| Public Member Functions inherited from TALMaSSObject | |
| void | OnArrayBoundsError () |
|  | TALMaSSObject () |
| virtual | ~TALMaSSObject () |

|  |  |
| --- | --- |
| Protected Member Functions | |
| virtual bool | OnFarmEvent (FarmToDo event) |
| virtual void | PesticideResponse (void) |
| virtual int | st\_Developing () |
| virtual void | st\_Dying () |
| virtual void | st\_Maturing () |
| Protected Member Functions inherited from Skylark\_Base | |
| bool | DailyMortality (int mort) |
| Protected Member Functions inherited from TAnimal | |
| void | CorrectWrapRound (void) |
|  | Corrects wrap around co-ordinate problems. |

|  |  |
| --- | --- |
| Protected Attributes | |
| Skylark\_Male \* | m\_Dad |
| double | m\_EM |
| int | m\_EM\_fail |
| double | m\_GrNeed |
| int | m\_NestLeavingChance |
| bool | Sex |

|  |  |
| --- | --- |
| Additional Inherited Members | |
| Public Attributes inherited from Skylark\_Base | |
| int | Age |
| int | m\_Born\_x |
| int | m\_Born\_y |
| TTypesOfSkState | m\_CurrentSkState |
| int | m\_MyHome |
|  | The vegetation type where the skylark was born. |
| Skylark\_Population\_Manager \* | m\_OurPopulationManager |
| SkTerritories \* | m\_OurTerritories |
| double | m\_pcide\_conc |
| double | m\_pesticide\_accumulation |
| double | m\_Size |

---

## Constructor & Destructor Documentation

|  |  |  |  |
| --- | --- | --- | --- |
| Skylark\_Nestling::Skylark\_Nestling | ( | int | *x*, |
|  |  | int | *y*, |
|  |  | Skylark\_Male \* | *Daddy*, |
|  |  | Landscape \* | *L*, |
|  |  | SkTerritories \* | *Terrs*, |
|  |  | Skylark\_Population\_Manager \* | *SPM*, |
|  |  | int | *bx*, |
|  |  | int | *by*, |
|  |  | int | *mh* |
|  | ) |  |  |

References Skylark\_Base::Age, m\_Dad, m\_EM, m\_EM\_fail, m\_GrNeed, m\_NestLeavingChance, Skylark\_Base::m\_Size, MeanHatchingWeight, random(), Sex, CfgInt::value(), and CfgFloat::value().

: Skylark\_Base( x, y, Terrs, L, SPM, bx, by, mh ) {

m\_Dad = Daddy;

//Growth = 0;

// Must choose sex

if ( random( 2 ) == 1 ) Sex = true; else

Sex = false; // M or F

m\_Size = MeanHatchingWeight + ( ( ( random( 25 ) - 11 ) \* MeanHatchingWeight ) / 1000.0 );

Age = 1;

m\_EM\_fail = 0;

// From Skylarks Energy Calcs Mar05.xls

m\_EM = 0.3058 \* m\_Size + 0.5221;

m\_GrNeed = cfg\_PEmax.value(); // No of Kcals possible to use for growth on day 1

m\_NestLeavingChance = cfg\_NestLeavingChance.value();

}

---

## Member Function Documentation

|  |  |  |  |  |  |
| --- | --- | --- | --- | --- | --- |
| void Skylark\_Nestling::BeginStep | ( | void |  | ) |  |

virtual

Reimplemented from TAnimal.

Reimplemented in Skylark\_PreFledgeling.

References TAnimal::CheckManagement(), and TALMaSSObject::CurrentStateNo.

{

// If you are not dead already find out if you are killed today by agriculture

if ( CurrentStateNo != -1 ) CheckManagement();

}

|  |  |  |  |  |  |
| --- | --- | --- | --- | --- | --- |
| void Skylark\_Nestling::EndStep | ( | void |  | ) |  |

virtual

Reimplemented from TAnimal.

Reimplemented in Skylark\_PreFledgeling.

References TALMaSSObject::CurrentStateNo, Calendar::Date(), g\_date, Skylark\_Base::m\_CurrentSkState, m\_Dad, Skylark\_Male::OnNestPredatation(), PesticideResponse(), st\_Developing(), st\_Dying(), st\_Maturing(), TALMaSSObject::StepDone, toss\_Destroy, toss\_NDeveloping, toss\_NDying, and Landscape::Warn().

{

#ifdef \_\_CJTDebug\_5

if ( IsAlive() != 0x0DEADC0DE ) DEADCODEError();

#endif

if ( m\_CurrentSkState == toss\_Destroy ) return;

#ifdef \_\_CJTDebug\_5

if ( Dad )

if ( !Dad->DoIExistN( this ) ) {

g\_land->Warn( "Skylark\_Nestling::EndStep - Do I ExistN ", NULL ); exit( 1 );

}

#endif

switch ( m\_CurrentSkState ) {

case toss\_NDeveloping: // Develop

switch ( st\_Developing() ) {

case 3:

if ( m\_Dad != NULL ) m\_Dad->OnNestPredatation(); // Kill the whole brood

break;

case 2:

#ifdef TEST\_ISSUE\_DEATH\_WARRANT

printf( "Skylark\_Nestling::EndStep() : st\_Developing() %d\n", ( int )g\_date->Date() );

#endif

m\_CurrentSkState = toss\_NDying; // die

break;

case 1:

// Maturing must be called here because it happens the same day as develop

st\_Maturing(); // Do Maturing

m\_CurrentSkState = toss\_Destroy;

CurrentStateNo = -1;

StepDone = true;

break;

}

break;

case toss\_NDying: // Dying

st\_Dying();

StepDone = true;

break;

default:

/\* char errornum[20]; sprintf(errornum, "%d", m\_CurrentSkState );

g\_land->Warn("Skylark\_Nestling::EndStep(): Unknown state: ", errornum); exit(0); \*/

break;

}

#ifdef \_\_PESTICIDE\_RA

PesticideResponse();

#endif

}

|  |  |  |  |  |  |
| --- | --- | --- | --- | --- | --- |
| double Skylark\_Nestling::On\_FoodSupply | ( | double | *food* | ) |  |

virtual

Reimplemented from Skylark\_Base.

References Skylark\_Base::Age, CE\_nest, m\_EM, m\_GrNeed, and Skylark\_Base::m\_Size.

Referenced by Skylark\_Male::st\_CaringForYoung().

{

#ifdef \_\_CJTDebug\_5

if ( IsAlive() != 0xDEADC0DE )

DEADCODEError();

#endif

if ( m\_EM > 0 ) // Must maintain this

{

if ( a\_food > m\_EM ) {

a\_food -= m\_EM;

m\_EM = 0;

} else {

m\_EM -= a\_food;

a\_food = 0;

}

}

if ( m\_GrNeed > a\_food ) {

m\_GrNeed -= a\_food;

// Does the growing here

m\_Size += a\_food \* CE\_nest[ Age ]; // Conv Eff is g dw insect to g ww bird

a\_food = 0;

} else {

// Does the growing here at the maximum allowed rate

m\_Size += m\_GrNeed \* CE\_nest[ Age ]; // Conv Eff is g dw insect to g ww bird

a\_food -= m\_GrNeed;

m\_GrNeed = 0;

}

return a\_food;

}

|  |  |  |  |  |
| --- | --- | --- | --- | --- |
| void Skylark\_Nestling::OnDadDead | ( |  | ) |  |

References m\_Dad.

Referenced by Skylark\_Male::OnMateDying(), Skylark\_Male::OnMateLeaving(), Skylark\_Male::st\_Dying(), and Skylark\_Male::st\_ScaringOffChicks().

{

#ifdef \_\_CJTDebug\_5

if ( IsAlive() != 0xDEADC0DE )

DEADCODEError();

#endif

// Dad is dead - so must not send him a message

// So forget who he is (messaging to Dad must be checked for NULL on sending)

m\_Dad = NULL;

}

|  |  |  |  |  |
| --- | --- | --- | --- | --- |
| void Skylark\_Nestling::OnDeserted | ( |  | ) |  |

References TALMaSSObject::CurrentStateNo, Skylark\_Base::m\_CurrentSkState, TAnimal::m\_OurLandscape, Skylark\_Base::m\_OurPopulationManager, Landscape::SupplyDayInYear(), toss\_Destroy, and Skylark\_Population\_Manager::WriteSKPOM2().

Referenced by Skylark\_Male::OnBroodDesertion().

{

// Nobody to tell, nothing to do but vanish as soon as possible

m\_CurrentSkState = toss\_Destroy;

CurrentStateNo = -1;

#ifdef \_\_SKPOM

m\_OurPopulationManager->WriteSKPOM2(m\_OurLandscape->SupplyDayInYear(),2005);

#endif

}

|  |  |  |  |  |  |
| --- | --- | --- | --- | --- | --- |
| bool Skylark\_Nestling::OnFarmEvent | ( | FarmToDo | *event* | ) |  |

protectedvirtual

Reimplemented from TAnimal.

Reimplemented in Skylark\_PreFledgeling.

References Skylark\_Base::AddStriglingMort(), autumn\_harrow, autumn\_or\_spring\_plough, autumn\_plough, autumn\_roll, autumn\_sow, burn\_straw\_stubble, cattle\_out, cattle\_out\_low, cut\_to\_hay, cut\_to\_silage, cut\_weeds, Calendar::Date(), deep\_ploughing, Landscape::EventtypeToString(), fa\_ammoniumsulphate, fa\_greenmanure, fa\_manure, fa\_npk, fa\_pk, fa\_sludge, fa\_slurry, fp\_greenmanure, fp\_liquidNH3, fp\_manganesesulphate, fp\_manure, fp\_npk, fp\_npks, fp\_pk, fp\_sludge, fp\_slurry, fungicide\_treat, g\_date, glyphosate, growth\_regulator, harvest, hay\_bailing, hay\_turning, herbicide\_treat, hilling\_up, insecticide\_treat, Skylark\_Base::m\_CurrentSkState, TAnimal::m\_Location\_x, TAnimal::m\_Location\_y, TAnimal::m\_OurLandscape, molluscicide, mow, pigs\_out, product\_treat, random(), row\_cultivation, sleep\_all\_day, spring\_harrow, spring\_plough, spring\_roll, spring\_sow, straw\_chopping, strigling, strigling\_sow, stubble\_harrowing, Landscape::SupplyGrazingPressure(), swathing, syninsecticide\_treat, toss\_NDying, trial\_insecticidetreat, CfgInt::value(), Landscape::Warn(), water, and winter\_plough.

{

switch ( event ) {

case sleep\_all\_day:

case fp\_npks:

case fp\_npk:

case fp\_pk:

case fp\_manganesesulphate:

case fp\_manure:

case fp\_greenmanure:

case fp\_sludge:

case fa\_npk:

case fa\_pk:

case fa\_ammoniumsulphate:

case fa\_manure:

case fa\_sludge:

case herbicide\_treat:

case growth\_regulator:

case fungicide\_treat:

case insecticide\_treat:

case trial\_insecticidetreat:

case molluscicide:

case water:

case hay\_bailing:

case cut\_weeds:

break;

case autumn\_harrow:

case autumn\_roll:

case autumn\_sow:

case winter\_plough:

case deep\_ploughing:

case spring\_plough:

case spring\_harrow:

case spring\_roll:

case spring\_sow:

case autumn\_plough:

case fp\_liquidNH3:

case fa\_greenmanure:

case row\_cultivation:

case hilling\_up:

case harvest:

case cut\_to\_hay:

case cut\_to\_silage:

case straw\_chopping:

case hay\_turning:

case stubble\_harrowing:

case autumn\_or\_spring\_plough:

case burn\_straw\_stubble:

case mow:

#ifdef TEST\_ISSUE\_DEATH\_WARRANT

printf( "Skylark\_Nestling::OnFarmEvent() : %d : %d\n", event, ( int )g\_date->Date() );

#endif

m\_CurrentSkState = toss\_NDying;

break;

case fp\_slurry:

case fa\_slurry:

case swathing:

if ( random( 100 ) < 2 ) {

#ifdef TEST\_ISSUE\_DEATH\_WARRANT

printf( "Skylark\_Nestling::OnFarmEvent() : %d : %d\n", event, ( int )g\_date->Date() );

#endif

m\_CurrentSkState = toss\_NDying;

}

break;

case syninsecticide\_treat:

if ( random( 1000 ) < cfg\_insecticide\_direct\_mortN.value() ) {

#ifdef TEST\_ISSUE\_DEATH\_WARRANT

printf( "Skylark\_Nestling::OnFarmEvent() : %d : %d\n", event, ( int )g\_date->Date() );

#endif

m\_CurrentSkState = toss\_NDying;

}

break;

case strigling:

#ifndef \_\_NoStriglingEffect

if ( random( 100 ) < cfg\_strigling\_nestling.value() ) {

#ifdef TEST\_ISSUE\_DEATH\_WARRANT

printf( "Skylark\_Nestling::OnFarmEvent() : %d : %d\n", event, ( int )g\_date->Date() );

#endif

m\_CurrentSkState = toss\_NDying;

AddStriglingMort( 2 );

}

#endif

break;

case cattle\_out:

if ( m\_OurLandscape->SupplyGrazingPressure( m\_Location\_x, m\_Location\_y ) )

if ( random( 1000 ) < 20 ) {

#ifdef TEST\_ISSUE\_DEATH\_WARRANT

printf( "Skylark\_Nestling::OnFarmEvent() : %d : %d\n", event, ( int )g\_date->Date() );

#endif

m\_CurrentSkState = toss\_NDying;

}

break;

case cattle\_out\_low:

if ( m\_OurLandscape->SupplyGrazingPressure( m\_Location\_x, m\_Location\_y ) )

if ( random( 1000 ) < 5 ) {

#ifdef TEST\_ISSUE\_DEATH\_WARRANT

printf( "Skylark\_Nestling::OnFarmEvent() : %d : %d\n", event, ( int )g\_date->Date() );

#endif

m\_CurrentSkState = toss\_NDying;

}

break;

case pigs\_out:

#ifndef \_\_NoPigsOutEffect

if ( random( 100 ) < 20 ) {

#ifdef TEST\_ISSUE\_DEATH\_WARRANT

printf( "Skylark\_Nestling::OnFarmEvent() : %d : %d\n", event, ( int )g\_date->Date() );

#endif

m\_CurrentSkState = toss\_NDying;

}

#endif

break;

case strigling\_sow:

#ifndef \_\_NoStriglingEffect

if ( random( 100 ) < cfg\_strigling\_nestling.value() ) {

#ifdef TEST\_ISSUE\_DEATH\_WARRANT

printf( "Skylark\_Nestling::OnFarmEvent() : %d : %d\n", event, ( int )g\_date->Date() );

#endif

m\_CurrentSkState = toss\_NDying;

AddStriglingMort( 2 );

}

#endif

break;

case product\_treat:

break;

case glyphosate:

break;

default:

g\_land->Warn( "Skylark\_Nestling::OnFarmEvent(): ""Unknown event type:", m\_OurLandscape->EventtypeToString( event ) );

exit( 1 );

}

if ( m\_CurrentSkState == toss\_NDying ) return true; else

return false;

}

|  |  |  |  |  |
| --- | --- | --- | --- | --- |
| void Skylark\_Nestling::OnYouHaveBeenEaten | ( |  | ) |  |

References TALMaSSObject::CurrentStateNo, Skylark\_Base::m\_CurrentSkState, TAnimal::m\_OurLandscape, Skylark\_Base::m\_OurPopulationManager, Landscape::SupplyDayInYear(), toss\_Destroy, and Skylark\_Population\_Manager::WriteSKPOM2().

Referenced by Skylark\_Male::OnNestPredatation().

{

// Nobody to tell, nothing to do but vanish as soon as possible

m\_CurrentSkState = toss\_Destroy;

CurrentStateNo = -1;

#ifdef \_\_SKPOM

m\_OurPopulationManager->WriteSKPOM2(m\_OurLandscape->SupplyDayInYear(),2001);

#endif

}

|  |  |  |  |  |  |
| --- | --- | --- | --- | --- | --- |
| virtual void Skylark\_Nestling::PesticideResponse | ( | void |  | ) |  |

protectedvirtual

Reimplemented from Skylark\_Base.

Reimplemented in Skylark\_PreFledgeling.

Referenced by EndStep().

|  |  |  |  |  |
| --- | --- | --- | --- | --- |
| int Skylark\_Nestling::st\_Developing | ( |  | ) |  |

protectedvirtual

Reimplemented in Skylark\_PreFledgeling.

References Skylark\_Base::Age, Skylark\_Base::DailyMortality(), EM\_nest\_T, m\_EM, m\_GrNeed, m\_NestLeavingChance, TAnimal::m\_OurLandscape, Skylark\_Base::m\_OurPopulationManager, Skylark\_Base::m\_Size, NestLeavingWeight, NestlingMortProb, random(), Landscape::SupplyDayInYear(), Landscape::SupplyTemp(), CfgFloat::value(), and Skylark\_Population\_Manager::WriteSKPOM2().

Referenced by EndStep().

{

// On entry GrNeed is set to the remaining food that could have been eaten

// today

#ifdef \_\_CJTDebug\_5

if ( IsAlive() != 0x0DEADC0DE ) DEADCODEError();

#endif

// Timing of this function call is at the last moment of each day

if ( DailyMortality( NestlingMortProb ) ) {

return 3; //send brood eaten to Dad, will call OnYouHaveBeenEaten for all nestlings

}

// EM will be 0 if enough food has been given

/\*

if ( m\_EM > 0 )

{

if ( m\_EM\_fail > 2 )

{

#ifdef \_\_SKPOM

m\_OurPopulationManager->WriteSKPOM2( m\_OurLandscape->SupplyDayInYear(), 2002 );

#endif

return 2; //TransferToState(Die) Did not get EM for 2 days

}

} else

{

m\_EM\_fail = 0; // got EM so unset flag

}

\*/

if ( Age >= 6 )

{

if ( m\_Size >= NestLeavingWeight )

{

if ( random( 100 ) < m\_NestLeavingChance ) {

#ifdef \_\_SKPOM

m\_OurPopulationManager->WriteSKPOM2( m\_OurLandscape->SupplyDayInYear(), Age );

#endif

return 1;

} else

m\_NestLeavingChance \*= 4;

}

}

if ( Age == 11 )

{

#ifdef \_\_SKPOM

m\_OurPopulationManager->WriteSKPOM2( m\_OurLandscape->SupplyDayInYear(), 2004 );

#endif

return 2; // Taken too long so die

}

Age++; // age

// From Skylarks Energy Calcs Mar05.xls

if ( Age < 6 ) m\_EM = 0.31 \* m\_Size + 0.52; else

m\_EM = 0.24 \* m\_Size + 6.83;

// Below is the correction for thermoregulation (Below 4days this is really a

// female cost - but it is subtracted here instead).

// Assumes that 50% of the time (night) that the chicks are covered and at 36)

// EM += EM\_nest\_T[ Age ] \* ( 36 - ( ( 0.5 \* m\_OurLandscape->SupplyTemp() ) + 18 ) );

m\_EM += EM\_nest\_T[ Age ] \* ( 36 - ( m\_OurLandscape->SupplyTemp() ) );

m\_GrNeed = cfg\_PEmax.value();

return 0; // still growing and not dead

}

|  |  |  |  |  |
| --- | --- | --- | --- | --- |
| void Skylark\_Nestling::st\_Dying | ( |  | ) |  |

protectedvirtual

Reimplemented in Skylark\_PreFledgeling.

References TALMaSSObject::CurrentStateNo, Skylark\_Base::m\_CurrentSkState, m\_Dad, Skylark\_Male::OnNestlingDeath(), and toss\_Destroy.

Referenced by EndStep().

{

#ifdef \_\_CJTDebug\_5

if ( IsAlive() != 0x0DEADC0DE ) DEADCODEError();

#endif

// Timing: Zero time

// Only called before object is destroyed

// Checks that Dad exists

if ( m\_Dad != NULL ) m\_Dad->OnNestlingDeath( this );

m\_CurrentSkState = toss\_Destroy; // will kill it at end of step!

CurrentStateNo = -1;

}

|  |  |  |  |  |
| --- | --- | --- | --- | --- |
| void Skylark\_Nestling::st\_Maturing | ( |  | ) |  |

protectedvirtual

Reimplemented in Skylark\_PreFledgeling.

References PreFledgeling\_struct::age, Skylark\_Base::Age, Skylark\_struct::bx, Skylark\_struct::by, Skylark\_Population\_Manager::CreateObjects(), PreFledgeling\_struct::Dad, Skylark\_struct::L, Skylark\_Base::m\_Born\_x, Skylark\_Base::m\_Born\_y, m\_Dad, TAnimal::m\_Location\_x, TAnimal::m\_Location\_y, Skylark\_Base::m\_MyHome, TAnimal::m\_OurLandscape, Skylark\_Base::m\_OurPopulationManager, Skylark\_Base::m\_OurTerritories, Skylark\_Base::m\_Size, Skylark\_struct::mh, PreFledgeling\_struct::sex, Sex, PreFledgeling\_struct::size, Skylark\_struct::Terrs, Skylark\_struct::x, and Skylark\_struct::y.

Referenced by EndStep().

{

#ifdef \_\_CJTDebug\_5

if ( IsAlive() != 0x0DEADC0DE ) DEADCODEError();

#endif

if ( !m\_Dad ) return; // If Dad is gone then something went wrong and we died

// Timing: Occurs at 1 minute to midnight

// When created they must update dads pointer

PreFledgeling\_struct \* fps;

fps = new PreFledgeling\_struct;

fps->x = m\_Location\_x;

fps->y = m\_Location\_y;

fps->size = m\_Size;

fps->Dad = m\_Dad;

fps->age = Age;

fps->L = m\_OurLandscape;

fps->Terrs = m\_OurTerritories;

fps->sex = Sex;

fps->bx = m\_Born\_x;

fps->by = m\_Born\_y;

fps->mh = m\_MyHome;

m\_OurPopulationManager->CreateObjects( 2, this, NULL, fps, 1 );

delete fps;

// m\_OurPopulationManager will destroy object

}

|  |  |  |  |  |  |
| --- | --- | --- | --- | --- | --- |
| void Skylark\_Nestling::Step | ( | void |  | ) |  |

virtual

Reimplemented from TAnimal.

Reimplemented in Skylark\_PreFledgeling.

References Skylark\_Base::m\_CurrentSkState, TALMaSSObject::StepDone, toss\_Destroy, toss\_Initiation, toss\_NDeveloping, toss\_NDying, and Landscape::Warn().

{

#ifdef \_\_CJTDebug\_5

if ( IsAlive() != 0x0DEADC0DE ) DEADCODEError();

#endif

if ( StepDone ) return;

switch ( m\_CurrentSkState ) {

case toss\_Initiation: // Initial

m\_CurrentSkState = toss\_NDeveloping;

StepDone = true;

break;

case toss\_Destroy:

StepDone = true;

break;

case toss\_NDeveloping: // Develop

StepDone = true;

break;

case toss\_NDying: // Dying

StepDone = true;

break;

default:

g\_land->Warn( "Skylark\_Nestling::Step(): Unknown State ", NULL ); exit( 1 );

}

}

---

## Member Data Documentation

Skylark\_Male\* Skylark\_Nestling::m\_Dad

protected

Referenced by EndStep(), OnDadDead(), Skylark\_Nestling(), st\_Dying(), Skylark\_PreFledgeling::st\_Dying(), st\_Maturing(), and Skylark\_PreFledgeling::st\_Maturing().

double Skylark\_Nestling::m\_EM

protected

Referenced by On\_FoodSupply(), Skylark\_Nestling(), Skylark\_PreFledgeling::Skylark\_PreFledgeling(), st\_Developing(), and Skylark\_PreFledgeling::st\_Developing().

int Skylark\_Nestling::m\_EM\_fail

protected

Referenced by Skylark\_Nestling().

double Skylark\_Nestling::m\_GrNeed

protected

Referenced by On\_FoodSupply(), Skylark\_Nestling(), Skylark\_PreFledgeling::Skylark\_PreFledgeling(), st\_Developing(), and Skylark\_PreFledgeling::st\_Developing().

int Skylark\_Nestling::m\_NestLeavingChance

protected

Referenced by Skylark\_Nestling(), and st\_Developing().

bool Skylark\_Nestling::Sex

protected

Referenced by Skylark\_Nestling(), Skylark\_PreFledgeling::Skylark\_PreFledgeling(), st\_Maturing(), and Skylark\_PreFledgeling::st\_Maturing().

---

The documentation for this class was generated from the following files:

- skylarks\_all.h
- skylarks\_all.cpp


- Skylark\_Nestling
- Generated on Thu Jan 10 2013 13:15:38 for ALMaSS Skylark ODdox by
   1.8.1.1
